# On the Errors of Certain Published Opinions Concerning the Nature of the Malignant Cholera, and on the Treatment of That Complaint in Its Collapsed Stage by Single Grain Doses of Calomel

**Published:** 1849-11

**Authors:** 


*On the Errors of certain published Opinions Concerning the Nature of the Malignant Cholera, and on the Treatment of that Complaint in its Collapsed Stage by Single Grain Doses of Calomel.* By JOSEPH AYRE, M. D., Hull; Member of the Royal College of Physicians, London, and formerly Physician to the Hull General Infirmary.

In a communication from Mr. W. Home Popham, giving an account of the treatment pursued by him in the infant poor-house at Tooting, there is a reference made to me, and to the treatment I recommended in the malignant cholera, and a quotation given from some writing of mine on the subject. With regard to the treatment, I owe my thanks for the candor with which he has stated the discrepancy between his treatment and mine, he having given only half a grain of calomel every half-hour, in cases where I should have advised a grain every five minutes; I must, however, at the same time assure him that the use he made of calomel was no modification of my treatment, and his unsuccess has caused me no surprise. With respect to the quotation, I owe it also to that gentleman to say, that believing, as I could not fail to do, that he had fallen into an error in the hurry of writing, and that he had misquoted me, I learnt from him in answer to an application on the subject, that he had accidentally done so. The error of itself would be of little account in ordinary circumstances, but at the present moment it is of importance that it should be corrected, as involving a principle in relation to the *modus operandi* of calomel in the cholera, which I must disclaim as directly opposed to the views I have inculcated, and to the purpose for which I advocate the use of that medicine. In the passage quoted, I am made to state that I gave the calomel every five minutes, and in single grain doses, that it might be retained in the system, whereas it may be seen, by reference to my letters in the Lancet*, as well as to my work on the disease, that my sole purpose in giving calomel in a single grain dose, and repeated, pending the continuance of the collapse, every five minutes, combining a drop of laudanum with each dose, was to retain the medicine, and prevent its being either rejected from the stomach, or hurried through the bowels. In large doses, the calomel acts as an irritant to the stomach and bowels, and in the cholera, when so taken, it is rarely retained, but in the moderate dose of one grain with a drop of laudanum, and repeated continuously every five minutes, it is readily retained, and thus it becomes a most effective remedy to the stage of collapse, and by subduing it, a remedy to the whole complaint. To give the calomel, however, with a view to its being taken into the system, would be a practice meriting the severest condemnation, because, were it to be absorbed, a salivation would inevitably ensue. My recommendation of the treatment is limited to the stage of collapse, and every case that I had and have referred to was in that stage; and notwithstanding the large quantity of calomel which I found it necessary to give to very young children, I never had one child in the least degree affected with any soreness of the mouth, or with any other inconvenience from the medicine.

When gentlemen writing on the subject of cholera inform us that they give large doses of calomel in the stage of collapse, with a view to its being absorbed, and of acting upon the disease through the circulation, and even object to the use of small and frequently repeated doses, they wholly overlook the important fact, that pending the duration of the stage of collapse, no absorption takes place, or if it does, that no mercurial action ensues from it; for looking at the very large quantities that I and others have given in that stage, and without any mercurial action, it is impossible to imagine that any absorption occurs. The action, therefore, is on the stomach, and it is for the twofold purpose of having the medicine retained there, and of its effects to be prolonged there, that the small and frequent dose is employed. There may be those who object to the opinion I have inculcated on the mode in which it acts in subduing the disease—namely, by its restoring the secretion of the bile, and thus removing the venous congestion of the liver and the other organs whose circulation is associated with it. I have no overweening preference for any theory regarding the pathological condition forming the essence of the disease, or of the mode by which the medicine acts. It is sufficient for the purpose to know, that given in the manner adopted, and so earnestly recommended by me—that calomel (and it is advisedly that I assert it) so given, is a specific for the cure of the stage of collapse, and will prove such in the hands of all who will scrupulously employ it, according to the rule prescribed. But though

---

* *Ante*, Jan., Feb., March.

quite ready to abandon any theory regarding the pathology of the disease, upon having one more feasible shown to me, yet in answer to those who contend that there is no interruption to the secretion of the bile, as denoted by the large quantity of that fluid found in the gall-bladder, I would remind those persons, that it is most obvious that no bile is secreted pending the duration of the collapse, as is proved by two constant conditions—namely, that the discharges from the stomach and bowels are colorless, and this for the whole time that the collapse continues, and which may be two or three days, and that neither during that period, nor after the symptoms of collapse have passed away, is there even the least tinge of yellow in the skin. If, however, there was a secretion of the bile going on during this time,—and none of it, as is clearly the case, is discharged into the alimentary canal,—that absorption of the bile, and a jaundice as its consequence, would ensue. It is true, indeed, that the gall bladder is found full of a fluid, but it is much darker and more viscid than healthy bile; and it is quite conceivable that the first action of the morbid cause leading to the congestion of the liver may be to occasion temporarily a morbid increase of the secretory action, to be followed by its cessation, and next by the congestion as the result of it. When there is bile in the discharges, there is no congestion and no collapse,—when the discharges become colorless, the congestion is present, and the collapse has commenced; and however long may be the duration of the rice-colored evacuations, no jaundice ensues. To my mind it is conclusive, that pending the duration of the collapse, there is an absolute interruption to the secretion of the liver, and that calomel, as an acknowledged and universally admitted agent for promoting its secretion, is the efficient remedy for the congestion which results from the interruption of it. The question, however, in truth, is, not whether calomel given in small doses, acts in this or that manner, but whether its action is beneficial; for though its mode of acting be held forever as unknown, we shall be in no worse condition than we are, in regard to quinine or arsenic in the cure of intermittents; and if experience in its use instructs us aright in using it, so that it cures, we have obtained for the sufferers, in relation to its use, all that we need.

But to the error which has prevailed concerning the assumed absorption of calomel in the stage of collapse, and of its operating thus through the system upon the disease, and to the fear that the quantity of the medicine to be taken would lead to salivation, I may here notice, as a further hindrance to the adoption of the treatment, the belief that the stage of collapse is irremediable, from the immense loss which is assumed to take place from the blood by the copious evacuations from the stomach and bowels. If it were true, as I have heard it contended for by one of the ablest of our writers on physiology, that the fluids discharged consist mainly of the serum of the blood, and that such loss from the system must render collapse irremediable, and that all endeavors, by calomel or other means, to remedy the evil, must prove unavailing, I would ask, what is to be said to the fact, which I and others have constantly witnessed, of persons whose evacuations have been enormous, recovering in a few days to their accustomed state of health, and even nearly of strength, and without the least signs of having sustained any sensible loss from the blood. The truth is, the fluids discharged are secreted fluids, and though *from* the blood, cannot be *of* the blood, for they occasion none of the effects which a hundredth part of them in blood would produce. To suffer, therefore, so loose and wholly untenable an opinion as this to become a hindrance to our just endeavors to save, as I fear it often has been, is making but a perverted use of our knowledge, such as it is, and leaping in the dark to the most fatal conclusions.

But next to the fatal error of concluding from a view of the discharges, that the collapsed stage is beyond the reach of a remedy, is the evil of the notion held by some writers, that cholera is essentially a febrile disease, and analogous to, if not identical with, ague, and that as emetics or bleeding, in some cases, and stimulants in others, or all these in succession, are the appropriate means for the cold stage of ague, so are they also in the blue, or what is falsely called the cold stage of cholera. That such opinions are held is certainly to me a matter of surprise, and looking to the names of Dr. Chambers, and Dr. Billing, who have advanced them, and to the evil practice which they may lead to, I feel myself called upon to combat them as wholly inconsistent with the facts we possess, both of the phenomena of the cholera and of the effects of medical treatment upon it.

In the Lectures on Cholera, by Dr. Chambers, published in late numbers of the Lancet, I observed that it is admitted that the malignant cholera is only an aggravated form of the European disorder of that name, and, in fact, that they acknowledge the same pathological conditions, the difference in the phenomena being dependent on a difference in the intensity of the proximate cause. Taking this fact to be granted, who, it may be asked, has ever seen the European cholera exhibit the symptoms of an intermittent? In the cold or collapsed stage of cholera there is no feeling of coldness on the part of the patient, though with a death-like coldness of the skin, whilst in the ague patient there is the most distressing sense of it, with little or no coldness on the surface; and whilst one desires to have external heat applied, the other is oppressed by it. In the paroxysm of ague, the perspiration succeeds the fever as this does the cold stage, but the moisture on the surface is a part of the cold stage of cholera, and not its sequence. Ague is essentially a febrile complaint, and so rarely stopped at its first paroxysm, that we may predicate of it, that an individual attacked by it will have a succession of paroxysms before he is fully cured; but of the cholera, whether mild or malignant, one cold stage suffices, and if he recovers from the first paroxysm he has no second attack of it.

But there are some still more important and conclusive distinctions to be noticed between cholera and ague and every other form of fever, and which, if I mistake not, will set the question at rest forever. The distinctions I refer to consist in the different, and even opposite treatment which each respectively requires for its cure.

In the two lectures by Dr. Chambers, just alluded to, and which, it appears, were delivered in February, 1832, and when, I presume, that gentleman could know little or nothing practically of the complaint, there are some speculations on the proximate cause of the disorder, or its pathological condition, which a better acquaintance with the phenomena of the disease should have corrected. There are also indications of cure laid down, based upon the view taken of its nature, and which correspond to the course pursued with such lamentable unsuccess through the whole period of that epidemic, and, as it should seem, from the reports given of its mortality, with no less zeal and unsuccess in the present one.

It would be beside my purpose, which is to advocate one line of treatment, were I to enter at length into an examination of the merits of any other; and passing by therefore, the multifarious, and, as it seems to me, most incongruous and opposite means of cure recommended in collapse, and none of which are adapted for the cold stage of ague, I shall content myself with remarking, that in representing calomel to be a sedative, and to be employed with that intent in the stage of collapse, and in the dose of ten or twenty grains every three or four hours, is an error both hypothetically and practically of no common amount. It is true that a grain of calomel, given at bedtime to a patient laboring under a functional derangement of the liver, and whose rest had been broken by it, will experience a sedative effect from the medicine, even to the degree of believing that his rest had been given him by an opiate; but in this case it is hardly needful to say, the sedative operation results from the medicine correcting disordered functions; and calomel, therefore, whether in a large or small dose, is not a sedative in the sense in which that term is to be understood. But assuming that calomel acts *quo sedative*, what is there in the stage of collapse that calls for such a remedy, for excepting the cramps, which are not always violent and scarcely ever present at all in children near the infant age, there is no such disturbance of the nervous system as calls for the aid of a sedative; indeed, a state the very opposite of nervous excitement or irritability prevails, and whilst children never cry or manifest any other sign of impatience under their affliction, so adults, though quite sensible, exhibit a manifest indifference to their condition, and to what will be the the issue of it. But what is to be the value we are to set upon a medicine as a remedy, which, when taken in a large dose, is rejected almost immediately from the stomach, and which dose is recommended to be repeated every three or four hours, in a disease whose term is to be measured by minutes, and whose fatal course will often terminate before the time comes round for a second exhibition of the medicine?

But in whatever way calomel acts in subduing the stage of collapse, and whatever may be the suitable dose and times of its repetition, there is a distinction in regard to it, as applied to its use in the collapse of cholera, on the one hand; and to the cold stage of intermittents and remittents, on the other; that abundantly manifests an absolute dissimilarity in the pathological conditions of these complaints respectively, and in the treatment required for them; for whilst calomel would be not only pernicious, in the paroxysm of an intermittent, it is an efficient, and, with the exception of a drop of laudanum combined with each dose, is the only needed remedy for cholera; and when so employed, as I shall again briefly point out, is, in every sense of the word, a specific for the complaint. This is not newly uttered, nor unadvisedly; and under the indulgent favor of giving admission, in the pages of the Lancet, to this lengthy communication,—the last, I hope, I shall have to make,—I shall proceed to show statistically, that calomel, in single grain doses given every five or ten minutes, according to the intensity of the collapse, and with a drop of laudanum with each *for a given time*, is capable of restoring a patient to health without the intervention of any fever, and without the aid of any auxiliary means whatever. And here let me repeat what I stated in a former paper, that from the first to the last case of the disease which came under my care, embracing more than two hundred, it was a rule I constantly observed, not to hazard the safety of my conclusion regarding the power of calomel to remove the collapse, by any admixture of means; for having ascertained the power of that medicine to subdue the severest form of the disease, I neither bled nor gave stimulants, nor applied external heat or rubefacients to the surface. I only gave calomel as I have described, and with no other effect of any kind but that of speedily removing the collapse; and with the removal of the collapse, restoring at once the patient to health. To the care which I gave to simplify the treatment, I added that of having it witnessed; and beside the hospital assistants, I procured three medical friends, especially to visit and witness my cases of collapse, with a view to report their condition, and the results of the treatment. Of the 178 patients whom I cured out of 200, on whom I had an opportunity of trying the treatment, the average duration of the collapse in the whole who recovered did not exceed four days; that very few cases occurred where there was any soreness of the mouth, and not one in the least degree severely so; that in the few instances in which it occurred, it was generally with those whose collapse was the least intense, and where the least amount of calomel was taken, and that consecutive fever was of very rare occurrence among those who recovered, and always to be traced to some neglect or indiscretion on the part of the patient.

From the view which I shall now give, in a tabular form, of the cases of collapse, the number of days during which the patient was under treatment will be seen; and with respect to them, I must observe that no medicine was given, or other means used, beyond the time stated, and that the patient was cured and reported as such to the inspector of the local board of health. In regard to details, I may state that, with one exception, I uniformly gave the calomel in the form of a pill, which was compounded with a minute quantity of gum arabic, and made so small as to weigh, when dry, about one grain and a quarter. I always carried a large number about me, and made a memorandum of the number I left with each patient, and counted them at my next visit, by which I ascertained with what punctuality they had been taken. With the pills I left also a given number of drops of laudanum, with directions to add a given number of teaspoonfuls of water, and to give each pill in a teaspoonful of such mixture. The thirst of the patients, and their desire to have cold drink, made the teaspoonful of fluid most acceptable to them, and by being passed far into the mouth, the pill was readily floated down with the water.

To those who may take the trouble to examine the subjoined table, it will be shown that, greatly as salivation, with all its evil effects, might be apprehended, from the enormous quantity of calomel that was given to each patient, but four instances occurred amongst fifty cases, and that in so slight a degree, as not to merit notice, or any treatment for its cure. In fact, the inconvenience, such as it was, and it merited no other name, only occurred in a very few cases out of upwards of 176 patients who were recovered out of the stage of collapse; and with respect to it, it deserves to be noted, that it happened chiefly with those whose collapse was the least intense at the commencement of the treatment. With respect to the time given for the removal of the collapse, I desire it to be distinctly understood, that the recovery was not merely from the stage of collapse, but, strange and incredible as it may seem, to the condition and feeling of health, so that patients have been often seen by me, walking in a court or yard, or sitting at table with their family, who, four, three, or even two days before, were stretched on their bed, in a state of pulseless collapse.

In conclusion, I have only further to add, that, had I given in a tabular form the other cases which came under my care, amounting to nearly 130, and which underwent the same treatment, the same results would be seen—that the same amount of calomel was taken; and that with the exception of two or three patients whom I was called late to visit, and who fell into consecutive fever, the average duration of their complaint was under four days. As I have already intimated, I rely upon this being the last occasion on which I shall have to occupy your pages on these subjects, and shall bring to a close these observations, desultory as I fear they will be found; and at the risk of being chargeable with needless repetitions, will summarily restate:—

1st. That the malignant cholera is not essentially a febrile complaint, but may become so by its stage of collapse being unsubdued, when, if the patient survives it, an inflammatory action ensues in one or other of those tissues which were the seat of the congestion.

2nd. That the collapse becomes wholly removed, and the inflammatory reaction prevented, by a medicine whose acknowledged operation in most other cases is upon the liver, and as aiding the secretion of the bile; and that, on the removal of the collapse by calomel, and without the succession of fever, the appetite and other healthy conditions of the body are at once restored.

3d. That as no absorption of the calomel takes place pending the duration of the collapse, no inconvenience is sustained from the medicine, however large may be the amount taken; and that to enable the stomach to retain it, and to have its action prolonged upon it, it must be given in small and frequently-renewed doses, and with no other limit to the quantity than the duration of the collapse prescribes.

4th. That calomel so given to patients in every other known disease, would infallibly destroy them, from the absorption of the medicine which would take place, and would have no effect in subduing or removing any of those states which are severally assumed to exist in the collapse of cholera, as constituting its proximate cause, whether such states are held to be a corrupted state of the blood; or a morbid and spinal irritation of the ganglionic plexus; or an abeyance of the vital powers, from a loss of blood by the rice-colored discharges; or the direct action of a poisonous miasm on the brain and vertebral column.

5th, and lastly. That the treatment of collapse by small doses of calomel, as already described, and without the intervention of stimulants or other auxiliary means, and only assisted for a limited time with single drops of laudanum, is the lone means required, and that, when so used, it leads to a speedy remission of the severer symptoms of collapse, and by the removal of the collapse, brings the patient, in two, three, or four days, from a state of absolute and even pulseless prostration, to one of entire convalescence. *London Lancet.**

---

*Case of a Negro woman, who gave birth to twins of different color.* B. R. Carter, M.D. Virginia. (Communicated in a letter to one of the Editors.) I promised you when I left Philadelphia, that on reaching home, I would

* The table referred to is omitted.—Editor Buff. Med. Jour.

